# Positive Regulation of Acetate in Adipocyte Differentiation and Lipid Deposition in Obese Mice

**DOI:** 10.3390/nu15173736

**Published:** 2023-08-25

**Authors:** Changbao Sun, Ang Li, Huan Wang, Jiage Ma, Juncai Hou

**Affiliations:** 1 College of Food and Biological Engineering, Qiqihar University, Qiqihar 161006, China; sunchangbao1987@163.com (C.S.); liang621liang@163.com (A.L.); 2 College of Food Science, Northeast Agricultural University, Harbin 150030, China; jiage_ma@neau.edu.cn; 3 Branch of Animal Husbandry and Veterinary of Heilongjiang Academy of Agricultural Sciences, Qiqihar 161005, China; whuan05172023@163.com

**Keywords:** obesity, lipid metabolism, acetate, adipocyte differentiation, lipid deposition

## Abstract

Acetate is associated with adipocyte differentiation and lipid deposition. To further develop this scientific point, obese mice on a high-fat diet were given an intragastric administration of acetate for 8 weeks and mouse adipose mesenchymal stem cells (mAMSCs) were treated with acetate for 24 h. The results showed that the body weight, food intake, Lee’s index, adipose tissue coefficient, liver index, blood lipid levels, insulin resistance, pro-inflammatory factors levels and fatty lesions in liver and adipose tissue in obese mice treated with acetate increased markedly, while anti-inflammatory factors levels and liver function decreased significantly (*p* < 0.05). The mRNA expression levels of PPAR-γ, C/EBP-α, SREBP, AFABP, FAS, ACC-1, SCD-1, LPL, LEPR, GPR41 and GPR43 genes in adipose tissue and mAMSCs were significantly increased, while the mRNA expression levels of HSL, CPT-1, CPT-2, AMPK, AdipoR1 and AdipoR2 genes were significantly reduced (*p* < 0.05). Except for AMPK-α signaling pathway proteins, the phosphorylation levels of p38 MAPK, ERK1/2, JNK and mTOR were significantly increased (*p* < 0.05) and these changes were dose-dependent. The findings indicated that acetate played a positive role in regulating adipocyte differentiation and lipid deposition by activating MAPKs and mTOR signaling pathways (the expression up-regulation of genes such as PPAR-γ, C/EBP-α and SREBP-1, etc.) and inhibiting the AMPK signaling pathway (the expression down-regulation of genes such as HSL, CPT-1 and AMPK-α, etc.).

## 1. Introduction

In recent years, the incidence of obesity has gradually increased, and obesity has become a serious global issue for human health [1,2]. Obesity means that the body absorbs more energy than it expends, leading to excessive energy storage in white adipose tissue rather than brown adipose tissue. It has characteristics such as weight gain, elevated blood lipids, insulin resistance, inflammatory reactions, white adipose tissue hypertrophy and white adipocyte proliferation. In addition to secreting cytokines (such as leptin and adiponectin), the main function of white adipocytes is to store energy in the form of triglycerides in the cell when there is excess energy and release it in the event of an energy deficiency, while brown adipocytes exist in a very small amount in the body and plays a role in thermogenesis. Evidence has shown that short-chain fatty acids (SCFAs) are the main energy source and energy regulator of the body [3,4,5].

SCFAs are the primary metabolites produced via the gut microbial fermentation of non-digestible carbohydrates in the colon, mainly including acetate, propionate and butyrate, and the proportion of them was about 60:25:15, of which there was the highest content of acetate [6,7,8]. SCFAs are predominantly metabolized in the liver and colon epithelium and play a crucial role in maintaining metabolic as an energy source for the host. Butyrate is the main energy source for intestinal epithelial cells and regulates their proliferation; propionate is transported to the liver where it is used as a substrate for hepatic gluconeogenesis, maintaining energy balance and glucose tolerance; acetate is readily absorbed into the portal vein and used as a substrate for lipid and cholesterol synthesis in the liver [9,10,11]. It was found that acetate could regulate the expression of genes related to lipid metabolism by binding to short-chain fatty acid receptors GPR41 and GPR43 [12], which indicated that acetate had an important impact on adipocyte differentiation, lipid deposition and insulin resistance. For example, Perry et al. [13] found that obese mice on a high-fat diet had a higher level of acetate in serum and feces; meanwhile, the body weight and insulin secretion of obese mice injected subcutaneously with acetate further increased significantly. On the contrary, Fan et al. [14] reported that the body weight and blood lipid level of obese mice were significantly reduced after 5 weeks of the intragastric administration of acetate, and the mRNA expression levels of fatty acid decomposing factors (CPT1 and CPT2) in adipocytes were significantly increased. Therefore, based on this opposite scientific point, the current study further explored the effect of acetate on lipid metabolism in obese mice and its mechanism, which is of great significance for the treatment of obesity, insulin resistance and other related diseases.

## 2. Materials and Methods

### 2.1. Animal Studies

In the current study, 7-week-old male C57BL/6J mice (SPF) weighing 19.74 ± 0.75 g were purchased from Beijing Vital River Laboratory Animal Technology Co., Ltd. (Beijing, China), with an animal production license number of SCXK (Beijing) 2016-0006. The mice were independently housed in plastic cages in a standard animal laboratory under controlled conditions of constant temperature (24 ± 2 °C), constant humidity (50~60%) and a light/dark cycle for 12 h. During the experiment, mice were allowed to freely eat and drink water (filtered through a 0.22 μm filter). All mice were adaptively fed for two weeks. In the first week, mice were fed a general control low-fat feed (D12450B, fat/kcal% = 10). In the second week, 15 mice were randomly selected as the control group and continued to be fed a general control feed. Other mice were fed a high-fat feed (D12492, fat/kcal% = 60) to simulate obesity models. The low-fat feed (LFD) and high-fat feed (HFD) used in this study were both purchased from Beijing Keao Xieli Feed Co., Ltd. (Beijing, China).

After the two-week adaptation period, high-fat diet-mice were randomly divided into 4 groups, with 15 mice in each group, including high-fat-diet group 0 (HFD-0), high-fat-diet group 1 (HFD-1), high-fat-diet group 2 (HFD-2) and high-fat-diet group 3 (HFD-3). Mice in the HFD-1 group, HFD-2 group and HFD-3 group were gavaged daily with 0.2 mL of sodium acetate solution (Dalian Meilun Biological Technology Co., Ltd., Dalian, China) at concentrations of 1.0 g/kg, 2.0 g/kg and 3.0 g/kg, respectively. Mice in the control group (LFD-0) and HFD-0 group were gavaged daily with 0.2 mL of 0.9% NaCl solution (Dalian Meilun Biological Technology Co., Ltd., Dalian, China). The mice were gavaged continuously for 8 weeks and their body weight, length and food intake were measured at a fixed time per week throughout the protocol. The Lee’s index, adipose tissue coefficient and organ index were calculated according to the following formula [15]: Lee’s index = [body weight (g) × 1000] ^1/3^/length (cm), adipose tissue coefficient (%) = adipose tissue weight (g)/body weight (g) × 100, organ index = organ weight (g)/body weight (g) × 100.

### 2.2. Sample Collection

At the end of the gavage intervention experiment, mice were fasted for 12 h overnight, and blood samples were collected in centrifuge tubes by removing the eyeball. After being left for 2 h at a constant temperature of 37 °C, the serum was separated from the whole blood using an H1750R centrifugation instrument (Hunan Xiangyi Laboratory Instrument Development Co., Ltd., Changsha, China) at 4 °C and 3000 rpm for 10 min. The heart, liver, spleen, lungs, kidneys and adipose tissue (epididymal adipose, perirenal adipose and abdominal subcutaneous adipose) were quickly collected and weighed under sterile conditions after sacrifice. Some of them were put into 10% neutral buffered formalin solution (Merck Chemical Technology Co., Ltd., Shanghai, China) and the rest were snap-frozen in liquid nitrogen and then stored at −80 °C until further analysis.

### 2.3. Serum Bio-Parameter Analysis

The blood lipid (total cholesterol (TC), triglyceride (TG), low-density lipoprotein cholesterol (LDL-C) and high-density lipoprotein cholesterol (HDL-C)), liver function indicators (alanine aminotransferase (ALT), aspartate aminotransferase (AST), gamma-glutamyl transferase (GGT) and alkaline phosphatase (ALP)), adipose cytokines (fasting blood glucose (FBG) and insulin (INS)) levels were measured using the 7600-220 automated biochemical analyzer (Hitachi Co., Ltd., Tokyo, Japan). The adipose cytokines (leptin (LEP) and adiponectin (ADPN)) and inflammatory cytokine (interleukin-1β/6/4/10 (IL-1β/6/4/10) and tumor necrosis factor-α (TNF-α)) levels were measured via ELISA in accordance with the instructions of the test kit (Nanjing Jiancheng Bioengineering Institute, Nanjing, China).

### 2.4. Tissue Histology

#### 2.4.1. Hematoxylin–Eosin Staining

An analysis of tissue histology was conducted following the modified method as previously described [16,17]. For hematoxylin–eosin (H&E) staining; liver and abdominal subcutaneous adipose tissues fixed with 10% neutral buffered formalin solution (Merck Chemical Technology Co., Ltd., Shanghai, China) for 24 h were embedded in paraffin and cut into sections of 4~6 μm thickness using a vibratome (VT-1200S, Leica, Tokyo, Japan). The sections were deparaffinized in xylene (Shanghai Aladdin Bio-Chem Technology Co., Ltd., Shanghai, China), rehydrated using ethanol solution, stained with hematoxylin–eosin (Wuhan Baiqiandu Biotechnology Co., Ltd., Wuhan, China), and then dehydrated using gradient ethanol solution (70%, 80%, 90%, 95% and 100%). Eventually, the sections were embedded in paraffin and analyzed using a BZ-X710 all-in-one fluorescence microscope with the attached viewer software (Keyence Co., Ltd., Osaka, Japan).

#### 2.4.2. Oil Red O Staining

For Oil Red O staining, liver tissues fixed with 10% neutral buffered formalin solution (Merck Chemical Technology Co., Ltd., Shanghai, China) for 24 h were washed with water and dried, embedded in a frozen section medium 6502 (Thermo Fisher Scientific Co., Ltd., Shanghai, China), cut into sections of 4~6 μm thickness using a vibratome (VT-1200S, Leica, Tokyo, Japan) and stained with Oil Red O (Wuhan Baiqiandu Biotechnology Co., Ltd., Wuhan, China). Eventually, the sections were analyzed using a BZ-X710 all-in-one fluorescence microscope with the attached viewer software (Keyence Co., Ltd., Japan).

### 2.5. Real-Time PCR

The total RNA was extracted from abdominal subcutaneous adipose tissues and differentiated cells using a Trizol reagent kit (Tiangen Biochemical Technology Co., Ltd., Beijing, China) per the manufacturer’s protocol and transcribed into cDNA using Transcriptor cDNA Synth. Kit (Tiangen Biochemical Technology Co., Ltd., Beijing, China) in accordance with the instructions. Rt-PCR reactions were performed on a Step One Plus Rt-PCR detection system (ThermoFisher Scientific Co., Ltd., Shanghai, China) with the reaction processes: pre-denaturation at 95 °C for 10 s, followed by 40 cycles of 95 °C for 5 s and 60 °C for 34 s. The β-Actin rRNA gene was used as an endogenous control. The relative fold changes of the mRNA expression level of lipid metabolism-related genes were calculated in accordance with the ΔCt method [17]. Primer sequences are shown in Table 1.

### 2.6. Western Blotting

The abdominal subcutaneous adipose tissue was re-suspended in pre-cooled RIPA buffer and homogenized for one minute. The samples were incubated on ice for 30 min, followed by being centrifuged at 12,000 rpm for 10 min at 4 °C. The supernatants were collected, and the OD value was measured via ELISA at a wavelength of 570 nm in accordance with the instructions of the Bicinchoninic Acid protein concentration assay kit (Beyotime Biotechnology Co., Ltd., Beijing, China). The samples were denatured via heating. The target gene proteins (PPAR-γ, C/EBP-α, HSL, LPL, FAS, ACC-1, CPT-1, AMPK-α, GPR41 and GPR43) and phosphorylated signaling pathway proteins (p38 MAPK, ERK1/2, JNK, AMPK-α and mTOR) were separated via SDS polyacrylamide gel electrophoresis in accordance with Western blot methods [18]. After the blotting membranes were blocked with bovine serum albumin (Beyotime Biotechnology Co., Ltd., Beijing, China) at room temperature for 30 min, the membranes were incubated overnight at 4 °C with primary antibodies. Subsequently, the membranes were incubated with a secondary antibody for 2 h. The antibodies used in this study were purchased from Cell Signaling Technology, Danvers, MA, U.S.A. β-actin was used as the loading control. The blotting membranes developed a color with an enhanced chemiluminescence assay kit (Beyotime Biotechnology Co., Ltd., Beijing, China), and then we analyzed the grayscale of the target strip.

### 2.7. Cell Culture

The mouse adipose mesenchymal stem cells (Newgain Biotechnology Co., Ltd., Wuxi, China) were cultured following the modified method as previously described [19,20]. Briefly, the thawed cells were cultured in the original DMEM/F12 medium (containing 10% fetal bovine serum and 100 IU/mL of double antibody) under a humidified atmosphere containing 5% CO_2_ at 37 °C. Cells were grown to about 90% confluence over a 5- to 7-day culture period with medium changes every 2 days. Subsequently, cells were detached via standard trypsinization and transferred to a adipogenic differentiation DMEM/F12 medium (containing 1 μmol/L dexamethasone, 10 μg/mL of insulin, 200 μmol/L of indomethacin and 0.5 mmol/L of IBMX) for further adipocyte differentiation culture for 14 days with medium changes every 2 days. Cells collected on days 0, 3, 6, 9, and 12 of differentiation culture were investigated for lipid droplet deposition via Oil Red O staining and we measured the mRNA expression level of lipid metabolism-related genes via Rt-PCR. Acetate of different concentrations (0 mmol/L, 3 mmol/L, 6 mmol/L, and 9 mmol/L) was added to the adipogenic differentiation DMEM/F12 medium (Invitrogen, Carlsbad, CA, USA) with cell-differentiated culture for 3 days. Cells were collected after acetate treatment for 6 h, 12 h, and 24 h. Next, cells were investigated for lipid droplet deposition via Oil Red O staining and we measured the mRNA expression level of lipid metabolism-related genes via Rt-PCR.

### 2.8. Statistical Analysis

Statistical analyses were performed using SPSS 20.0 software. The descriptive statistics data are presented in the form of mean and standard deviation (Mean ± SD). Multiple comparisons were carried out via a one-way analysis of variance (ANOVA) with Tukey’s test, and *p* < 0.05 was considered a significant difference.

## 3. Results

### 3.1. Body Weight and Food Intake of Mice

**Figure 1:**
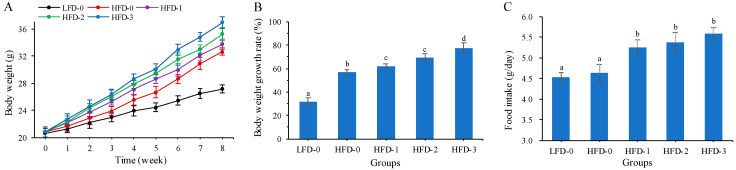
Body weight and food intake of mice in each group. Acetate promoted the elevation of (**A**) body weight, (**B**) body weight growth rate and (**C**) food intake in obese mice on a high-fat diet in a dose-dependent manner. Data are mean ± SD, and statistical significance was determined using a one-way analysis of variance (ANOVA) with Tukey’s multiple comparison test, *n* = 15/group. Different lowercase letters in Figure 1B indicate significant differences between groups (*p* < 0.05). LFD-0 represents control mice treated with 0.9% NaCl solution via gavage. HFD-0 represents obese mice treated with 0.9% NaCl solution via gavage. HFD-1 represents obese mice treated with 1.0 g/kg of acetate via gavage. HFD-2 represents obese mice treated with 2.0 g/kg of acetate via gavage. HFD-3 represents obese mice treated with 3.0 g/kg of acetate via gavage.

The body weight of mice on a high-fat diet exceeded 20% of the body weight of the control group, indicating that a high-fat diet successfully induced obesity in mice [21]. During the gavage intervention period, the body weight of all groups showed an upward trend (Figure 1A). The body weight of mice in the LFD-0 group increased from 20.64 ± 0.68 g to 27.15 ± 0.58 g, and the body weight of mice in the HFD-0 group increased from 20.85 ± 0.73 g to 31.57 ± 0.55 g, while the body weight of mice in the HFD-3 group increased from 20.85 ± 0.73 g to 36.97 ± 0.82 after the intragastric intervention with a high dose of acetate. The body weight of all high-fat-diet mice exceeded 20% that of the control group, indicating that the high-fat diet successfully induced obesity in mice. The body weight growth rates of HFD-1, HFD-2 and HFD-3 group mice were significantly higher than those of LFD-0 and HFD-0 group mice (*p* < 0.05), and the greater the dose of acetate, the higher the body weight growth rate of mice (Figure 1B). It was observed that the food intake of mice on a high-fat diet was higher than that of the control group (Figure 1C). The food intake of mice in the HFD-1, HFD-2 and HFD-3 groups was significantly higher than that of mice in the LFD-0 and HFD-0 groups (*p* < 0.05). The results showed that acetate gavage treatment promoted the body weight and food intake of obese mice on a high-fat diet in a dose-dependent manner.

### 3.2. Lee’s Index and Adipose Tissue Coefficient of Mice

**Figure 2:**
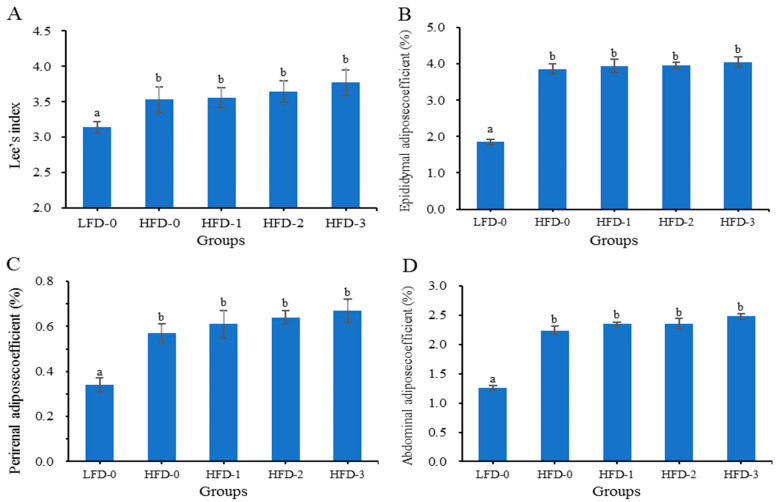
The Lee’s index and adipose tissue coefficient of mice in each group. Acetate promoted the elevation of the (**A**) Lee’s index, (**B**) epididymal adipose tissue coefficient, (**C**) perirenal adipose tissue coefficient and (**D**) abdominal subcutaneous adipose tissue coefficient in obese mice on a high-fat diet. Data are mean ± SD, and statistical significance was determined using a one-way analysis of variance (ANOVA) with Tukey’s multiple comparison test, *n* = 15/group. Different lowercase letters in figure indicate significant differences between groups (*p* < 0.05). LFD-0 represents control mice treated with 0.9% NaCl solution via gavage. HFD-0 represents obese mice treated with 0.9% NaCl solution via gavage. HFD-1 represents obese mice treated with 1.0 g/kg of acetate via gavage. HFD-2 represents obese mice treated with 2.0 g/kg of acetate via gavage. HFD-3 represents obese mice treated with 3.0 g/kg of acetate via gavage.

Lee’s index and the adipose tissue coefficient are effective indicators for evaluating the obesity degree in experimental animals [22,23]. Figure 2 shows that the Lee’s index and adipose tissue coefficient of mice on a high-fat diet (HFD-0, HFD-1, HFD-2 and HFD-3) were significantly higher than those of mice in the control group (LFD-0) (*p* < 0.05). The Lee’s index and adipose tissue coefficient of mice in the HFD-0, HFD-1, HFD-2 and HFD-3 groups gradually increased with the increase in the acetate gavage dosage, and there were no significant differences (*p* > 0.05). The results showed that a high-fat diet promoted lipid deposition and led to obesity in mice, and acetate further promoted lipid deposition in obese mice on a high-fat diet.

### 3.3. Organ Index of Mice

The organ index of mice on a high-fat diet (HFD-0, HFD-1, HFD-2 and HFD-3) was higher than that of mice in the control group (LFD-0), as shown in Table 2. The liver organ index of mice in HFD-0, HFD-1, HFD-2 and HFD-3 groups gradually increased with the increase in the acetate gavage dosage, and there were no significant differences (*p* > 0.05). The results indicated that acetate gavage treatment could further increase the body weight of high-fat-diet obese mice, but also increase the liver weight.

### 3.4. Serum Bio-Parameter Analysis

#### 3.4.1. Analysis of Blood Lipid Level

Blood lipids, mainly including total cholesterol (TC), triglyceride (TG), low-density lipoprotein cholesterol (LDL-C) and high-density lipoprotein cholesterol (HDL-C), could reflect whether or not the lipid metabolism of mice was abnormal. From Table 3, it could be seen that the TC, TG and LDL-C levels in all high-fat diet obese mice were significantly higher than those in the control group mice (*p* < 0.05), while the HDL-C level was significantly lower than that in the control group mice (*p* < 0.05). The HDL-C level (2.53 ± 0.03 mmol/L) of mice in the LFD-0 group was higher, while the TC level (2.94 ± 0.01 mmol/L), TG level (0.94 ± 0.01 mmol/L) and LDL-C level (0.95 ± 0.01 mmol/L) were lower. However, after inducing obesity with a high-fat diet, the HDL-C level (1.33 ± 0.01 mmol/L) of mice in the HFD-0 group was significantly reduced, while the TC level (4.79 ± 0.02 mmol/L), TG level (2.15 ± 0.03 mmol/L) and LDL-C level (1.93 ± 0.01 mmol/L) were significantly increased. The HDL-C level of obese mice treated with acetate via gavage gradually decreased, while the TC, TG and LDL-C levels gradually increased. The results showed that a high-fat diet could cause an increase in blood lipid levels in mice, leading to abnormal lipid metabolism. Acetate intragastric administration could further increase the blood lipid level in obese mice, inducing abnormal lipid metabolism in a dose-dependent manner.

#### 3.4.2. Analysis of Liver Function Indicator Levels

The liver function indicators are described in Table 4. It was found that the levels of AST, ALT, AKP, and GGT in the control group (LFD-0) were lower, indicating that the liver cells of mice may not have been damaged, and their liver function was normal. However, the levels of AST, ALT, AKP, and GGT in obese mice on a high-fat diet (HFD-0, HFD-1, HFD-2 and HFD-3) were significantly higher than those in the control group mice (*p* < 0.05). The AST/ALT ratio (0.89) of LFD-0 group mice was less than 1.00, while the AST/ALT ratios (1.05, 1.12, 1.57 and 1.60) of high-fat diet mice were greater than 1.00, indicating high-fat-diet-induced liver cell damage or inflammation in obese mice, leading to abnormal liver function. The AST, ALT, AKP and GGT levels of mice in the HFD-1, HFD-2 and HFD-3 groups gradually increased with the increase in the gavage dose of acetate. The results suggested that there was liver injury or inflammation in obese mice on a high-fat diet, and acetate intragastric administration aggravated liver injury or inflammation in obese mice on a high-fat diet.

#### 3.4.3. Analysis of Inflammatory Cytokine Levels

Inflammatory cytokines in serum include pro-inflammatory (IL-1β, IL-6 and TNF-α) and anti-inflammatory cytokines (IL-4 and IL-10). In Table 5, the levels of IL-1β, IL-6 and TNF-α in obese mice were significantly higher compared to those of the control group, while the levels of IL-4 and IL-10 were significantly lower than those in the control group (*p* < 0.05). After the intragastric treatment of acetate, the levels of IL-1β, IL-6 and TNF-α in HFD-1, HFD-2 and HFD-3 groups gradually increased and were higher than those in the HFD-0 group, while the levels of IL-4 and IL-10 in HFD-1, HFD-2, and HFD-3 groups decreased significantly and were lower compared to those of the HFD-0 group (*p* < 0.05). The results implicated that the pro-inflammatory cytokine levels of obese mice increased, and the anti-inflammatory cytokines levels decreased. The inflammatory response of obese mice on a high-fat diet was further aggravated via acetate gavage in a dose-dependent manner.

#### 3.4.4. Analysis of Adipose Cytokine Levels

**Figure 3:**
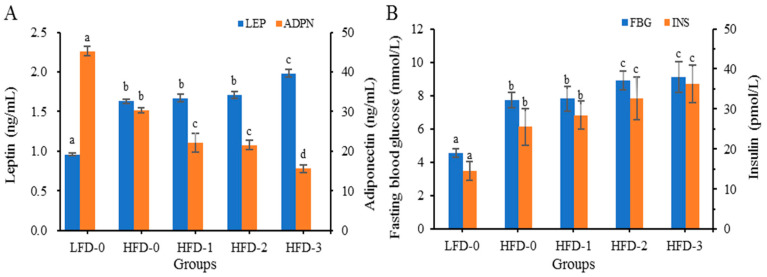
Serum leptin, adiponectin, fasting blood glucose and insulin levels of mice in each group. (**A**) Increased level of leptin and reduced level of adiponectin due to acetate treatment; (**B**) Increased levels of fasting blood glucose and insulin due to acetate treatment. Data are mean ± SD, and statistical significance was determined using a one-way analysis of variance (ANOVA) with Tukey’s multiple comparison test; *n* = 15/group. Different lowercase letters in the same color bars indicate significant differences between groups (*p* < 0.05). LFD-0 represents control mice treated with 0.9% NaCl solution via gavage. HFD-0 represents obese mice treated with 0.9% NaCl solution via gavage. HFD-1 represents obese mice treated with 1.0 g/kg of acetate via gavage. HFD-2 represents obese mice treated with 2.0 g/kg of acetate via gavage. HFD-3 represents obese mice treated with 3.0 g/kg of acetate via gavage.

The serum leptin (LEP), adiponectin (ADPN), fasting blood glucose (FBG) and insulin (INS) levels of mice in each group are depicted in Figure 3. Compared to those in the control group, obese mice had higher levels of LEP and lower levels of ADPN (Figure 3A). After intragastric treatment with acetate, the LEP levels of mice in the HFD-1, HFD-2 and HFD-3 groups were significantly higher than those of mice in the LFD-0 and HFD-0 groups, and the ADPN levels were significantly lower than those of mice in the LFD-0 and HFD-0 groups (*p* < 0.05). The LEP levels of mice in the HFD-3 group were significantly higher than those of mice in the HFD-1 and HFD-2 groups, while the ADPN levels were significantly lower than those of mice in the HFD-1 and HFD-2 groups (*p* < 0.05).

The FBG and INS levels of obese mice were higher than those of mice in the control group (Figure 3B). Compared with those in the LFD-0 group, the FBG levels and INS of mice in the HFD-0 group were significantly increased (*p* < 0.05). After intragastric treatment with acetate, the FBG and INS levels of mice in the HFD-2 and HFD-3 groups were markedly higher compared to those in of mice in the HFD-0 and HFD-1 groups (*p* < 0.05). The results indicated that a high-fat diet caused abnormal glucose metabolism in obese mice, reduced insulin sensitivity, and induced insulin resistance in obese mice. Acetate gavage treatment further reduced insulin sensitivity and aggravated insulin resistance in obese mice on a high-fat diet.

### 3.5. Analysis of Tissue Histology

**Figure 4:**
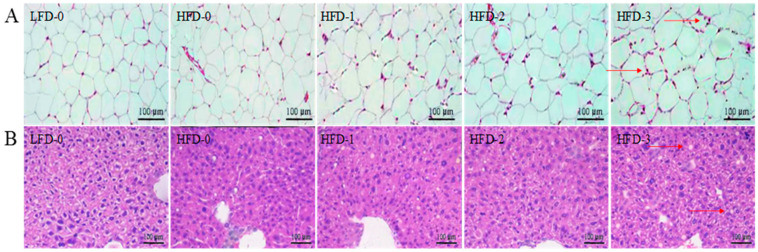
Hematoxylin–eosin staining of abdominal subcutaneous adipose tissue and liver tissue. (**A**) Increased white lipid accumulation and inflammatory response in the abdominal subcutaneous adipose tissue of obese mice due to acetate treatment, with the red arrow indicating coronal structure; (**B**) Increased degree of liver injury and fatty lesions in the liver tissue of obese mice due to acetate treatment, with the red arrow indicating lipid vacuoles. Magnification = 200×; scale bar = 100 μm. LFD-0 represents control mice treated with 0.9% NaCl solution via gavage. HFD-0 represents obese mice treated with 0.9% NaCl solution via gavage. HFD-1 represents obese mice treated with 1.0 g/kg of acetate via gavage. HFD-2 represents obese mice treated with 2.0 g/kg of acetate via gavage. HFD-3 represents obese mice treated with 3.0 g/kg of acetate via gavage.

The liver tissue and abdominal subcutaneous adipose tissue stained with H&E are illustrated in Figure 4. The abdominal subcutaneous adipose tissue structure of mice in the control group (LFD-0) was clear and complete with adipocytes in a circular or elliptical shape, evenly sized cells, and no inflammatory cell infiltration (Figure 4A). On the contrary, the abdominal subcutaneous adipose tissue structure of obese mice was fuzzy, the adipocytes were unevenly enlarged, some adipocytes were damaged, and a coronary structure was observed in the local stroma with the infiltration of macrophages, indicating that the abdominal subcutaneous adipose tissue cells were inflamed. With the increase in the acetate intragastric dose, the adipocytes of mice in the HFD-1, HFD-2, and HFD-3 groups became more significantly increased with more coronary structures and more inflammatory cell infiltration. The results showed that acetate could make obese mice accumulate more white fat and aggravate inflammatory reactions.

The liver tissue structure of mice in the control group (LFD-0) was clear and complete with clear boundaries, a neat arrangement and a uniform size, without fatty lesions (Figure 4B). Many white lipid vacuoles appeared in the liver tissue of obese mice with obvious fatty lesions. Small lipid droplets appeared in some liver cells, cell integrity was disrupted, cell content flowed out, and inflammatory cell infiltration was severe. Additionally, with the increase in the acetate intragastric dose, the more lipid vacuoles there were in the liver tissue of mice in the HFD-1, HFD-2 and HFD-3 groups, the more obvious the fatty lesions in liver cells were. The results indicated that acetate increased the body weight of obese mice, and also increased the degree of liver injury and fatty lesions.

**Figure 5:**
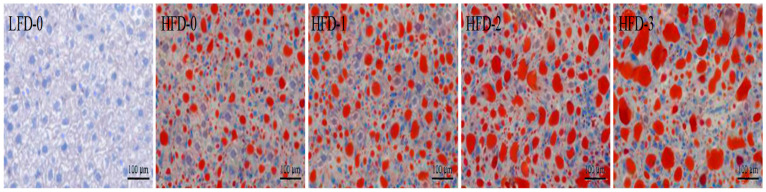
Increased lipid deposition in obese mice that was further aggravated via acetate gavage treatment indicated via the Oil Red O staining of liver tissue. Magnification = 200×; scale bar = 100 μm. LFD-0 represents control mice treated with 0.9% NaCl solution via gavage. HFD-0 represents obese mice treated with 0.9% NaCl solution via gavage. HFD-1 represents obese mice treated with 1.0 g/kg of acetate via gavage. HFD-2 represents obese mice treated with 2.0 g/kg of acetate via gavage. HFD-3 represents obese mice treated with 3.0 g/kg of acetate via gavage.

The liver tissue stained with Oil Red O is shown in Figure 5. It was observed that the control group (LFD-0) had an intact liver cell structure, clear boundaries, and no fatty degeneration or red lipid droplet deposition. Conversely, a large number of red lipid droplets appeared in the liver tissue of obese mice, with an incomplete cell structure and fatty lesions. With the increase in the acetate intragastric dose, the number and volume of red lipid droplets in the liver tissue of mice in the HFD-1, HFD-2, and HFD-3 groups gradually increased. The results suggested that there was a large amount of lipid deposition in the liver tissue of obese mice, and acetate aggravated lipid deposition in the liver tissue of obese mice.

### 3.6. Analysis of Relative mRNA Expression Levels of Lipid Metabolism-Related Genes in Adipose Tissue

**Figure 6:**
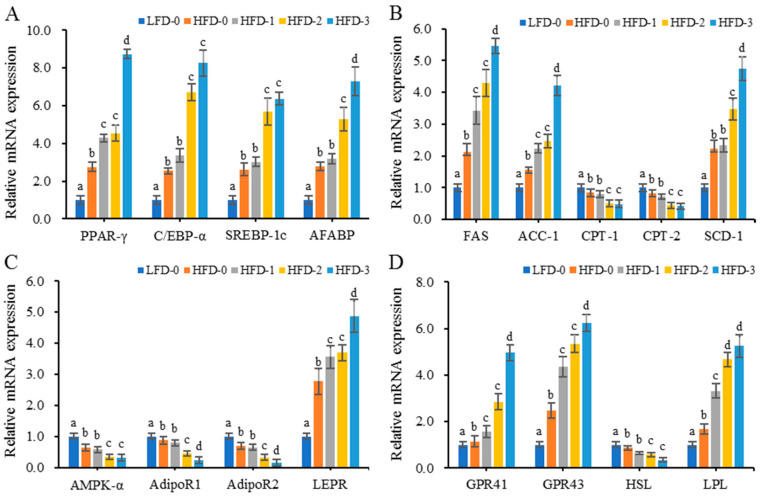
The relative mRNA expression levels of lipid metabolism-related genes in the abdominal subcutaneous adipose tissue of mice. (**A**) Increased relative mRNA expression levels of adipocyte differentiation factor (PPAR-γ, C/EBP, SREBP, and AFABP) genes due to acetate treatment; (**B**) Increased relative mRNA expression levels of fatty acid synthesis factor (FAS, ACC-1, and SCD-1) genes and decreased relative mRNA expression levels of fatty acid decomposition factor (CPT-1, and CPT-2) genes due to acetate treatment; (**C**) Increased relative mRNA expression level of leptin receptor (LEPR) gene and decreased relative mRNA expression levels of adiponectin receptor (AdipoR1, and AdipoR2) and AMPK-α genes due to acetate treatment; (**D**) Increased relative mRNA expression levels of short-chain fatty acid receptors (GPR41 and GPR43) and lipid synthesis factor (LPL) genes and decreased relative mRNA expression levels of the lipolytic factor (HSL) gene due to acetate treatment. Data are mean ± SD, and statistical significance was determined using one-way analysis of variance (ANOVA) with Tukey’s multiple comparison test; *n* = 15/group. Different lowercase letters in the same gene indicate significant differences between groups (*p* < 0.05). LFD-0 represents control mice treated with 0.9% NaCl solution via gavage. HFD-0 represents obese mice treated with 0.9% NaCl solution via gavage. HFD-1 represents obese mice treated with 1.0 g/kg of acetate via gavage. HFD-2 represents obese mice treated with 2.0 g/kg of acetate via gavage. HFD-3 represents obese mice treated with 3.0 g/kg of acetate via gavage.

The relative mRNA expression levels of lipid metabolism-related genes in abdominal subcutaneous adipose tissue are shown in Figure 6. The relative mRNA expression levels of adipocyte differentiation factor (PPAR-γ, C/EBP, SREBP, and AFABP), fatty acid synthesis factor (FAS, ACC-1, and SCD-1), leptin receptor (LEPR), lipid synthesis factor (LPL) and short-chain fatty acid receptor (GPR41 and GPR43) genes in obese mice were significantly higher than those in the control group (*p* < 0.05), while the relative mRNA expression levels of lipolytic factor (HSL), fatty acid decomposition factor (CPT-1 and CPT-2), adiponectin receptor (AdipoR1 and AdipoR2) and AMPK-α genes were significantly lower than those in the control group (*p* < 0.05). Acetate gavage treatment further reduced or increased the relative mRNA expression level of lipid metabolism-related genes in obese mice on a high-fat diet. The results showed that a high-fat diet could induce the upregulation of relative mRNA expression level of genes related to fatty acid synthesis in mice and promote lipid accumulation. Acetate intragastric treatment could promote the upregulation of the relative mRNA expression level of genes related to lipid synthesis and lipid accumulation in obese mice on a high-fat diet.

### 3.7. Analysis of Protein Expression Levels of Lipid Metabolism-Related Genes in Adipose Tissue

**Figure 7:**
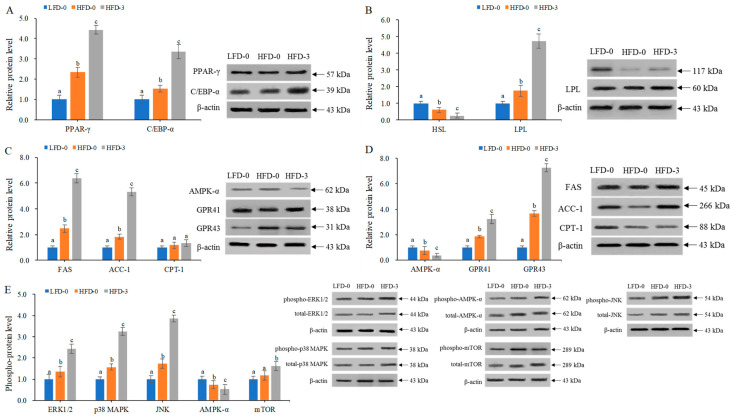
The protein expression levels of lipid metabolism-related genes in the abdominal subcutaneous adipose tissue of mice. (**A**) Increased protein expression levels of adipocyte differentiation factor (PPAR-γ and C/EBP-α) genes due to acetate treatment; (**B**) Increased protein expression level of the lipid synthesis (LPL) gene and decreased protein expression level of the lipolytic factor (HSL) gene due to acetate treatment; (**C**) Increased the protein expression levels of fatty acid synthesis factor (FAS and ACC-1) genes and decreased protein expression level of fatty acid decomposition factor (CPT-1) genes due to acetate treatment; (**D**) Increased protein expression levels of short-chain fatty acid receptor (GPR41 and GPR43) genes and decreased protein expression level of fatty acid metabolism signaling factors (AMPK-α) due to acetate treatment; (**E**) Increased phosphorylation levels of signaling pathway proteins (p38 MAPK, ERK1/2, JNK and mTOR) and decreased phosphorylation levels of signaling pathway proteins (AMPK-α) due to acetate treatment, with the phosphorylation levels being represented by phospho-protein/total protein. A representative Western blot is shown in the figure. Data are mean ± SD, and statistical significance was determined using a one-way analysis of variance (ANOVA) with Tukey’s multiple comparison test; *n* = 15/group. Different lowercase letters in the same gene/protein indicate significant differences between groups (*p* < 0.05). LFD-0 represents control mice treated with 0.9% NaCl solution via gavage. HFD-0 represents obese mice treated with 0.9% NaCl solution via gavage. HFD-3 represents obese mice treated with 3.0 g/kg of acetate via gavage.

This study found that acetate intragastric treatment could promote the mRNA expression levels of lipid metabolism-related genes and affect the levels of lipid metabolism in obese mice. Therefore, Western blotting was employed to determine the protein levels of lipid metabolism-related genes (PPAR-γ, C/EBP-α, HSL, LPL, FAS, ACC-1, CPT-1, AMPK-α, GPR41 and GPR43) and the phosphorylation levels of signaling pathway proteins (p38 MAPK, ERK1/2, JNK, AMPK-α and mTOR) in the abdominal subcutaneous adipose tissue of mice in the LFD-0, HFD-0 and HFD-3 groups, as shown in Figure 7. Compared with those in the control group, the protein expression levels of genes promoting lipid synthesis (PPAR-γ, C/EBP-α, FAS, ACC-1, LPL, GPR41 and GPR43) and the phosphorylation levels of signaling pathway proteins (p38 MAPK, ERK1/2, JNK and mTOR) were significantly increased in the abdominal subcutaneous adipose tissue of high-fat-diet-induced obese mice, while the protein expression levels of genes promoting lipid decomposition (HSL and AMPK-α) and their phosphorylation levels were significantly reduced (*p* < 0.05). After intragastric treatment with acetate, the protein levels of lipid metabolism-related genes in obese mice further increased or decreased. The results showed that acetate increased the phosphorylation levels of signaling pathway proteins (p38 MAPK, ERK1/2, JNK and mTOR) in the adipose tissue of obese mice but decreased the phosphorylation level of signaling pathway proteins (AMPK-α). Acetate activated MAPKs and the mTOR signaling pathway, inhibited the AMPK signaling pathway, regulated the expression levels of PPAR-γ, C/EBP-α, FAS, ACC-1, LPL, GPR41 and GPR43 genes, and promoted lipid deposition in obese mice on a high-fat diet.

### 3.8. Effect of Acetate on the Differentiation of Mouse Adipose Mesenchymal Stem Cells

**Figure 8:**
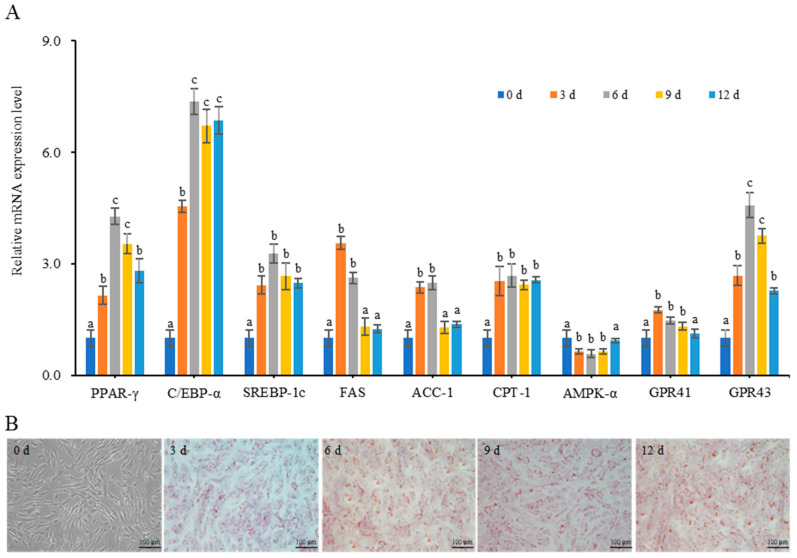
The relative mRNA expression levels of lipid metabolism-related genes and lipid droplet deposition in differentiated cells. (**A**) The relative mRNA expression levels of lipid synthesis-related genes that increased from the 3rd day of cell differentiation; data are mean ± SD, and statistical significance was determined using a one-way analysis of variance (ANOVA) with Tukey’s multiple comparison test; *n* = 15/group. Different lowercase letters in the same gene indicate significant differences between groups (*p* < 0.05). (**B**) Lipid droplets began to form in cells from the 3rd day of cell differentiation. Magnification = 200×; scale bar = 100 μm.

Cells collected on days 0, 3, 6, 9, and 12 of differentiation culture were investigated for lipid droplet deposition via Oil Red O staining and we measured the mRNA expression level of lipid metabolism-related genes via Rt-PCR, as shown in Figure 8. The relative mRNA levels of adipose differentiation factor (PPAR, C/EBP, and SREBP), fatty acid metabolism factor (FAS, ACC-1, and CPT-1) and SCFA receptors (GPR41 and GPR43) genes in cells significantly increased from the third day of cell differentiation, while the relative mRNA expression level of the AMPK-α gene significantly decreased from the third day of cell differentiation (*p* < 0.05) (Figure 8A). Mouse adipose mesenchymal stem cells that were not induced to undergo adipogenic differentiation showed spindle-shaped, single and dispersed adherent growth (Figure 8B). Starting from the third day of adipogenic differentiation, the cell morphology gradually transformed from a spindle to polygonal or elliptical shape, and there were lipid droplets of varying sizes in the cytoplasm. As the induction time extended, the lipid droplets gradually became larger and more numerous. Therefore, cells induced to differentiate for 3 days were used as a model for further analysis.

Three days after adipogenesis-induced differentiation, mouse adipose mesenchymal stem cells were treated with different concentrations of acetate. The relative mRNA expression levels of lipid metabolism-related genes are shown in Table 6. When treated with acetate for 6 h, 12 h and 24 h, compared with those of the control group, the relative mRNA expression levels of PPAR-γ, C/EBP-α and SREBP-1c genes increased significantly with the increase in the acetate concentration (*p* < 0.05). The relative mRNA expression levels of FAS, ACC-1, CPT-1 and AMPK-α genes decreased significantly (*p* < 0.05) after treatment with 3 mmol/L of acetate, but increased gradually after treatment with 6 mmol/L and 9 mmol/L of acetate; the relative mRNA expression levels of GPR41 and GPR43 genes increased with the increase in the acetate concentration, and the relative mRNA expression levels of GPR43 genes increased significantly (*p* < 0.05). The results showed that acetate treatment could promote the differentiation and lipid deposition of mouse adipose mesenchymal stem cells.

**Figure 9:**
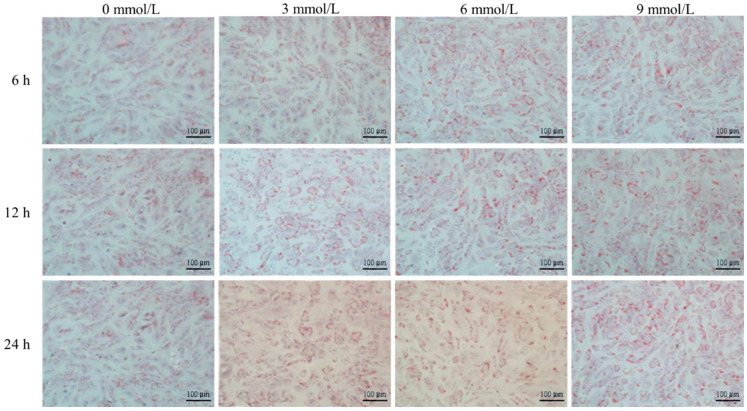
Oil Red O staining of mice adipose-derived mesenchymal stem cells treated with acetate indicating that acetate promoted the lipid deposition of cells in a dose-dependent manner. Magnification = 200×; scale bar = 100 μm.

The Oil Red O staining of mice adipose-derived mesenchymal stem cells treated with acetate is shown in Figure 9. After being treated with different concentrations of acetate for different times, the size of mouse adipose mesenchymal stem cells increased with the prolongation of the induced differentiation time and the increase in acetate concentration, and intracellular lipid droplets gradually increased and aggregated, showing a dose-dependent pattern.

## 4. Discussion

Obesity has become a serious global health issue, and is developed when energy absorption exceeds energy expenditure, resulting in extra energy being stored in adipose tissue in the form of triglycerides. This reduces the ability of lipid-oxidative metabolism to occur, and increases the inflammation development of adipose tissue, the secretion of pro-inflammatory adipokines and insulin resistance [3,24]. Evidence has shown that SCFAs affected adipose tissue metabolism [25], particularly acetate, which was the most abundant SCFAs in the body’s system. Therefore, this study explored the mechanism of acetate regulating lipid metabolism through the treatment of obese mice on a high-fat diet with acetate via gavage.

In addition to body weight, Lee’s index, the adipose tissue coefficient, and the organ index also indirectly reflected the obesity degree of mice [23]. This study found that the food intake, Lee’s index, adipose tissue coefficient and liver index of obese mice treated with acetate via gavage were higher than those of the control group in a dose-dependent manner, indicating that acetate improved the obesity degree of mice.

The large amount of lipids contained in the high-fat diet was digested by the intestine and entered the blood, causing an increase in the content of free fatty acids, promoting the synthesis of triglycerides, and leading to an increase in blood lipid levels. The clinical significance of determining blood lipids was the reflection of the level of the body’s lipid metabolism [26,27]. It was observed that the blood lipid levels in obese mice treated with acetate intragastric administration were increased in a dose-dependent manner, indicating that acetate aggravated abnormal lipid metabolism in obese mice.

The liver was the main site of lipid metabolism. When there is abnormal lipid metabolism in the body, liver tissue might suffer from liver injury or inflammation. The AST, ALT, AKP and GGT levels in serum were important indicators for clinical liver function testing [28]. We found that the AST, ALT, AKP and GGT levels in obese mice treated with a gavage of acetate gradually increased with the increase in the gavage dose of acetate, indicating that acetate aggravated liver injury and liver inflammation in obese mice.

Inflammatory cytokines are mainly secreted by peripheral immune cells (such as macrophages and lymphocytes), and work together with other cytokines to activate immune responses and mediate normal cellular activity in the body [29]. There are pro-inflammatory cytokines and anti-inflammatory cytokines in the inflammatory response. Pro-inflammatory cytokines (such as IL-1β, IL-6 and TNF-α) can activate various immune cells and promote inflammation, while anti-inflammatory cytokines (such as IL-4 and IL-10) and activate some other cells and reduce inflammatory reaction [30]. This study found that the pro-inflammatory cytokines levels in obese mice treated with acetate via gavage increased, and the anti-inflammatory cytokine level decreased in a dose-dependent manner, indicating that acetate aggravated the inflammatory response of high-fat-diet-induced obese mice.

In addition to inflammatory cytokines, adipose tissue also released adipocytokines and extracellular vesicles, with the most representative being LEP and ADPN. The levels of LEP and ADPN were closely related to the obesity degree, and an increase in adipose tissue often lead to an increase in LEP levels and a decrease in ADPN levels [31,32]. When the body experienced energy deficiency or decreased body lipids, the LEP level decreased, stimulating the body’s foraging behavior, and inhibiting insulin secretion to reduce energy consumption. On the contrary, when the body had excess energy or increased body lipids, the LEP level increased, inhibiting the body’s food intake, promoting insulin secretion, and accelerating lipid metabolism [33]. In addition, obese individuals had high levels of leptin, and were prone to the problem of leptin resistance with characteristics such as reduced satiety, and increased food intake and body weight, causing a delay in the satisfaction signal of the hunger hormone, leading to more food intake [34]. Different from that of LEP, the ADPN level decreased with the increase in adipose tissue. ADPN has metabolic and immune-related activities, can stimulate fatty acid oxidation, reduces the TG level, increases insulin sensitivity, inhibits inflammatory cytokines (IL-1β, IL-6 and TNF-α), and induces anti-inflammatory cytokines (IL-4, IL-10) [35]. Hyperleptin and hypoadiponectinemia caused by obesity might increase the inflammatory response [36]. This study found that the LEP level in obese mice treated with acetate via gavage increased and the ADPN level decreased in a dose-dependent manner, suggesting that acetate promoted lipid deposition in obese mice.

Fasting blood sugar and insulin levels truly reflect the body’s glucose metabolism ability and insulin sensitivity with the avoidance of the influence of diet or other factors. This study found that the fasting blood sugar and insulin levels of obese mice treated with acetate via gavage increased; this study’s results corresponded to the proposal of Perry et al. [13] who reported that acetate aggravated the abnormal glucose metabolism and insulin resistance of obese mice.

The pathological observation of liver tissue and abdominal subcutaneous adipose tissue also directly showed the effect of acetate on lipid metabolism in obese mice. In obese individuals, significant morphological changes occurred in adipose tissue, including an increase in adipocytes and macrophage aggregation. These changes lead to a more significant inflammatory state in adipose tissue, reflected by an increase in the secretion of pro-inflammatory mediators and a decrease in the secretion of insulin-sensitizing protein ADPN in adipose tissue. The increase in the inflammatory status of adipose tissue was believed to lead to the occurrence of systemic insulin resistance [37]. Macrophages aggregated and infiltrated into adipose tissue, accompanied by a more significant inflammatory state, presenting a pro-inflammatory phenotype, expressing more pro-inflammatory cytokines (IL-1β, IL-6 and TNF-α) and blocking the effect of insulin [38]. The liver tissue had also undergone significant morphological changes, with lipid droplets appearing in the liver tissue, forming many lipid voids, causing lipid deposition, and leading to liver function damage and tissue inflammation, which is consistent with the results of the serum AST, ALT, AKP, and GGT levels.

GPR41 and GPR43 are two short-chain fatty acid receptors confirmed in current research, which can be activated by SCFAs to regulate metabolism, immunity and other functions [39,40]. However, SCFAs have different activation effects on GPR41 and GPR43. The effects of GPR41 are as follows: valerate = propionate = butyrate > acetate > formate; those of GPR43 are as follows: acetate = propionate > butyrate > valerate = formate [41]. GPR41 and GPR43 were widely expressed in various tissue cells, such as adipose cells, immune cells, intestinal endocrine L cells, etc. After reaching specific tissues through blood circulation, acetate acted as a ligand to combine with GPR, triggering downstream effects, and regulating the expression of related genes and proteins. Acetate stimulated the secretion of LEP by activating GPR41 and GPR43 in adipose tissue, increasing the body’s food intake, and then promoting lipid deposition [42]. Lipid deposition was closely related to lipid oxidative decomposition and synthesis. Studies have shown that acetate could regulate a variety of cytokines in adipose tissue, inhibit the oxidative decomposition of fatty acids in the liver, and thus increase lipid deposition [43]. In this study, we found that acetate gavage treatment significantly increased the mRNA expression levels of adipocyte differentiation factor (PPAR-γ, C/EBP-α, SREBP-1c and AFABP), fatty acid synthesis factor (FAS, ACC-1 and SCD-1), leptin receptor (LEPR), fat synthesis factor (LPL), and short-chain fatty acid receptor (GPR41 and GPR43) genes, while the mRNA expression levels of adipolysis factor (HSL), fatty acid decomposition factor (CPT-1 and CPT-2), adiponectin receptor (AdipoR1 and AdipoR2) and fatty acid metabolism regulation signal protein (AMPK-α) genes were significantly decreased (*p* < 0.05). This study’s findings corresponded to the proposal of Huang et al. [44] who reported that the expression up-regulation of PPAR-γ, C/EBP-α and SREBP-1c genes could activate the expression of FAS, ACC-1 and SCD-1 genes, promoting the de novo synthesis of lipid in the liver and increasing lipid deposition in the liver and adipose tissue. The results indicated that acetate could promote lipid deposition, consistent with evidence from Zhao et al. [17] who reported that acetate derived from microbiota promoted hepatic lipogenesis. In the study of Wang et al. [45] suggested that the expression of the AMPK-α gene prevented the transfer of activated HSL to lipid droplets, thereby inhibiting the basal lipolysis of adipocytes.

The differentiation process of adipocytes was regulated by various cytokines, such as PPAR-γ, C/EBP-α, SREBP-1c and AMPK-α, etc. A study reported that acetate could promote the differentiation of 3T3-L1 preadipocytes in mice [46], which increased the expression levels of PPAR-γ, C/EBP-α and ERK1/2 [47,48]. Consistently, with the prolongation of the acetate treatment time, the mRNA expression levels of GPR41, GPR43, PPAR-γ, C/EBP-α and SREBP-1c genes of mouse adipose mesenchymal stem cells gradually increased, while the mRNA expression level of the AMPK-α gene gradually decreased. After being treated with different concentrations of acetate for different times, the size of mouse adipose mesenchymal stem cells increased with the prolongation of the induced differentiation time and the increase in the acetate concentration, and intracellular lipid droplets gradually increased in number and aggregated, showing a dose-dependent pattern. The results indicated that acetate promoted the differentiation and lipid deposition of mouse adipose mesenchymal stem cells.

By analyzing the protein expression levels of genes related to lipid metabolism in the adipose tissue of mice, it was found that the protein expression levels of genes promoting lipid synthesis (PPAR-γ, C/EBP-α, FAS, ACC-1, LPL, GPR41 and GPR43) and the phosphorylation levels of p38 MAPK, ERK1/2, JNK and mTOR signaling pathway proteins in obese mice treated with acetate were significantly increased, while the protein levels and phosphorylation levels of genes promoting lipid decomposition (HSL and AMPK-α) were significantly reduced (*p* < 0.05). It has been reported that acetate could increase the phosphorylation levels of p38 MAPK by activating GPR43, activating the adipocyte differentiation transcriptional regulation factor (PPAR-γ and C/EBP-α), and then promoting the differentiation of adipocytes [49,50]. Likewise, Kwon et al. [51] observed that suppressed protein levels of the phosphorylated MAPK-related factors ERK, JNK, and *p*-38 indicated the inhibition of PPAR-γ pathway-linked adipogenesis. The mTOR signaling pathway was also an important regulator of lipid synthesis metabolism, which could positively regulate the activity of lipid synthesis-related proteins. Some studies have shown that the activation of the mTOR pathway in 3T3-L1 preadipocytes could up-regulate the level of SREBP-1c, a transcription factor that promotes lipid synthesis, and promote lipid deposition while inhibiting the mTOR pathway could inhibit the de novo synthesis of lipids [52,53,54].

## 5. Conclusions

The findings indicate that acetate played a positive role in regulating adipocyte differentiation and lipid deposition by activating MAPKs and the mTOR signaling pathway (the expression up-regulation of genes such as PPAR-γ, C/EBP-α and SREBP-1, etc.) and inhibiting the AMPK signaling pathway (the expression down-regulation of genes such as HSL, CPT-1 and AMPK-α, etc.), which is of great significance for the treatment of obesity, insulin resistance and other related diseases.

## Figures and Tables

**Table 1:** Rt-PCR primer sequence information.

Gene	Forward (5′~3′)	Reverse (3′~5′)
β-Actin	F: GACCTCTATGCCAACACAGT	R: CACCAATCCACACAGAGTAC
PPAR-γ	F: GATGCACTGCTATGAGCACTT	R: AGAGGTCCACAGAGCTGATCC
C/EBP-α	F: GTCTACGCTCCACCACCATT	R: CCAAACCAGAAGGAAAGAGG
SREBP-1c	F: GATGTGCGAACTGGACA	R: CATAGGGGGCGTCAAACAG
AFABP	F: CAGATGACAGGAAAGGCAAG	R: TCCACCACCAGTTTATCACC
HSL	F: CATCCGGTCAGAGAGTACTTTT	R: TAGGGTTATGATGCTCTTCACC
LPL	F: CTGTTCCTCCAGTCGGTCAG	R: CGTCGCAGGAGGGAAGTTAG
FAS	F: CGGCGAGTCTATGCCACTAT	R: ACACAGGGACCGAGTAATGC
ACC-1	F: GGGAATACCTGTGGGAGTAGT	R: GCTGGATTATCTTGGCTTCA
CPT-1	F: ATGGTGGGCGACTAACT	R: TGCCTGCTGTCTGTGAG
CPT-2	F: ATGACCGTTTCTGCCATCC	R: AAGGTGTTGGTGTCGCTTCT
SCD-1	F: GCCATCATTATGAGTGCCAATT	R: AGGGATAAGAACGCTGAGAATT
AMPK-α	F: CCCTGTGTATGTGGCTCTG	R: GTGGGTGAACCTCTGCTT
AdipoR1	F: CGGCTCATCTACCTCTCCAT	R: ACACACCTGCTCTTGTCTGC
AdipoR2	F: CTGGCTCAAGGATAACGACTT	R: ATGTTGCCTGTCTCTGTGTG
LEPR	F: AAGAACAGAGATGAGGTGGTGC	R: CCAGTGTGGCGTATTTCACG
GPR41	F: CCATCTATCTCACCTCCCTGTTC	R: AACCAGCAGAGCCCACTGAC
GPR43	F: CGTCCAACTTCCGCTGGTA	R: CTTGTACTGCACGGGGTAGG

**Table 2:** Organ index of mice in each group.

Groups	Heart (%)	Liver (%)	Spleen (%)	Lungs (%)	Kidneys (%)
LFD-0	0.55 ± 0.01 ^a^	4.23 ± 0.21 ^a^	0.36 ± 0.01 ^a^	0.63 ± 0.01 ^a^	1.33 ± 0.08 ^a^
HFD-0	0.58 ± 0.02 ^a^	5.35 ± 0.26 ^b^	0.38 ± 0.02 ^a^	0.65 ± 0.01 ^a^	1.35 ± 0.07 ^a^
HFD-1	0.60 ± 0.01 ^a^	5.53 ± 0.32 ^b^	0.37 ± 0.01 ^a^	0.64 ± 0.03 ^a^	1.37 ± 0.05 ^a^
HFD-2	0.61 ± 0.01 ^a^	5.84 ± 0.24 ^b^	0.39 ± 0.02 ^a^	0.65 ± 0.02 ^a^	1.36 ± 0.06 ^a^
HFD-3	0.62 ± 0.01 ^a^	6.16 ± 0.27 ^b^	0.40 ± 0.02 ^a^	0.66 ± 0.02 ^a^	1.38 ± 0.08 ^a^

Data are mean ± SD, and statistical significance was determined using a one-way analysis of variance (ANOVA) with Tukey’s multiple comparison test, *n* = 15/group. Different superscript lowercase letters in the same column indicate significant differences in the organ index between groups (*p* < 0.05). LFD-0 represents control mice treated with 0.9% NaCl solution via gavage. HFD-0 represents obese mice treated with 0.9% NaCl solution via gavage. HFD-1 represents obese mice treated with 1.0 g/kg of acetate via gavage. HFD-2 represents obese mice treated with 2.0 g/kg of acetate via gavage. HFD-3 represents obese mice treated with 3.0 g/kg of acetate via gavage.

**Table 3:** Serum lipid level of mice in each group.

Groups	TC (mmol/L)	TG (mmol/L)	LDL-C (mmol/L)	HDL-C (mmol/L)
LFD-0	2.94 ± 0.01 ^a^	0.94 ± 0.01 ^a^	0.95 ± 0.01 ^a^	2.53 ± 0.03 ^a^
HFD-0	4.79 ± 0.02 ^b^	2.15 ± 0.03 ^b^	1.93 ± 0.01 ^b^	1.33 ± 0.01 ^b^
HFD-1	4.86 ± 0.04 ^b^	2.18 ± 0.01 ^b^	1.96 ± 0.02 ^b^	1.27 ± 0.01 ^b^
HFD-2	5.31 ± 0.02 ^bc^	2.41 ± 0.02 ^b^	2.42 ± 0.01 ^c^	0.97 ± 0.01 ^c^
HFD-3	5.94 ± 0.01 ^c^	2.57 ± 0.04 ^b^	2.54 ± 0.03 ^c^	0.82 ± 0.01 ^c^

Data are mean ± SD, and statistical significance was determined using a one-way analysis of variance (ANOVA) with Tukey’s multiple comparison test; *n* = 15/group. Different superscript lowercase letters in the same column indicate significant differences in serum lipid levels between groups (*p* < 0.05). LFD-0 represents control mice treated with 0.9% NaCl solution via gavage. HFD-0 represents obese mice treated with 0.9% NaCl solution via gavage. HFD-1 represents obese mice treated with 1.0 g/kg of acetate via gavage. HFD-2 represents obese mice treated with 2.0 g/kg of acetate via gavage. HFD-3 represents obese mice treated with 3.0 g/kg of acetate via gavage.

**Table 4:** Serum AST, ALT, AKP and GGT levels of mice in each group.

Groups	AST (U/L)	ALT (U/L)	AKP (U/L)	GGT (U/L)
LFD-0	28.76 ± 2.64 ^a^	32.25 ± 2.37 ^a^	55.47 ± 3.43 ^a^	13.22 ± 1.42 ^a^
HFD-0	67.43 ± 2.15 ^b^	64.27 ± 3.45 ^b^	80.15 ± 2.18 ^b^	44.28 ± 2.76 ^b^
HFD-1	83.28 ± 3.35 ^b^	74.58 ± 2.36 ^b^	93.49 ± 4.75 ^b^	62.21 ± 3.19 ^b^
HFD-2	135.47 ± 5.17 ^c^	86.41 ± 4.42 ^bc^	113.24 ± 4.36 ^c^	73.28 ± 4.35 ^bc^
HFD-3	153.43 ± 5.72 ^c^	95.78 ± 5.63 ^c^	135.28 ± 5.62 ^c^	86.45 ± 3.21 ^c^

Data are mean ± SD, and statistical significance was determined using a one-way analysis of variance (ANOVA) with Tukey’s multiple comparison test; *n* = 15/group. Different superscript lowercase letters in the same column indicate significant differences in liver function indicators between groups (*p* < 0.05). LFD-0 represents control mice treated with 0.9% NaCl solution via gavage. HFD-0 represents obese mice treated with 0.9% NaCl solution via gavage. HFD-1 represents obese mice treated with 1.0 g/kg of acetate via gavage. HFD-2 represents obese mice treated with 2.0 g/kg of acetate via gavage. HFD-3 represents obese mice treated with 3.0 g/kg of acetate via gavage.

**Table 5:** Serum inflammatory cytokines levels of mice in each group.

Groups	IL-1β (pg/mL)	IL-6 (pg/mL)	TNF-α (pg/mL)	IL-4 (pg/mL)	IL-10 (pg/mL)
LFD-0	13.53 ± 1.87 ^a^	15.17 ± 2.38 ^a^	78.64 ± 4.26 ^a^	51.36 ± 3.52 ^a^	93.57 ± 6.74 ^a^
HFD-0	30.72 ± 2.69 ^b^	27.61 ± 3.41 ^b^	138.15 ± 5.85 ^b^	30.15 ± 3.18 ^b^	45.19 ± 3.25 ^b^
HFD-1	31.24 ± 3.53 ^b^	28.54 ± 2.47 ^b^	141.63 ± 6.28 ^b^	23.55 ± 3.78 ^c^	24.43 ± 4.35 ^c^
HFD-2	37.67 ± 3.45 ^c^	35.53 ± 2.48 ^c^	145.47 ± 5.17 ^b^	21.47 ± 2.66 ^c^	23.25 ± 3.35 ^c^
HFD-3	39.26 ± 2.74 ^c^	36.29 ± 3.57 ^c^	166.43 ± 5.72 ^c^	12.24 ± 1.62 ^d^	11.36 ± 1.21 ^d^

Data are mean ± SD, and statistical significance was determined using a one-way analysis of variance (ANOVA) with Tukey’s multiple comparison test, *n* = 15/group. Different superscript lowercase letters in the same column indicate significant differences in serum inflammatory cytokine levels between groups (*p* < 0.05). LFD-0 represents control mice treated with 0.9% NaCl solution via gavage. HFD-0 represents obese mice treated with 0.9% NaCl solution via gavage. HFD-1 represents obese mice treated with 1.0 g/kg of acetate via gavage. HFD-2 represents obese mice treated with 2.0 g/kg of acetate via gavage. HFD-3 represents obese mice treated with 3.0 g/kg of acetate via gavage.

**Table 6:** Effect of acetate treatment on the relative mRNA expression levels of genes related to lipid metabolism in mice adipose-derived mesenchymal stem cells.

Time	Genes	0 mmol/L	3 mmol/L	6 mmol/L	9 mmol/L
6 h	PPAR-γ	1.00 ± 0.03 ^a^	1.51 ± 0.07 ^b^	1.45 ± 0.08 ^b^	1.43 ± 0.13 ^b^
	C/EBP-α	1.00 ± 0.07 ^a^	1.34 ± 0.05 ^b^	1.63 ± 0.08 ^c^	1.69 ± 0.15 ^c^
	SREBP-1 c	1.00 ± 0.08 ^a^	1.23 ± 0.05 ^b^	1.58 ± 0.09 ^c^	1.28 ± 0.10 ^b^
	FAS	1.00 ± 0.05 ^a^	0.70 ± 0.04 ^b^	0.93 ± 0.06 ^a^	1.05 ± 0.03 ^a^
	ACC-1	1.00 ± 0.03 ^a^	0.73 ± 0.03 ^b^	1.06 ± 0.08 ^a^	1.07 ± 0.06 ^a^
	CPT-1	1.00 ± 0.04 ^a^	0.83 ± 0.05 ^b^	1.06 ± 0.07 ^a^	1.08 ± 0.09 ^a^
	GPR41	1.00 ± 0.05 ^a^	1.09 ± 0.06 ^a^	1.33 ± 0.12 ^b^	1.36 ± 0.13 ^b^
	GPR43	1.00 ± 0.08 ^a^	1.26 ± 0.15 ^b^	1.57 ± 0.13 ^c^	1.87 ± 0.18 ^d^
	AMPK-α	1.00 ± 0.06 ^a^	0.77 ± 0.03 ^b^	1.03 ± 0.07 ^a^	1.05 ± 0.06 ^a^
12 h	PPAR-γ	1.11 ± 0.04 ^a^	1.31 ± 0.05 ^b^	1.55 ± 0.07 ^c^	1.63 ± 0.09 ^c^
	C/EBP-α	1.05 ± 0.08 ^a^	1.25 ± 0.04 ^b^	1.48 ± 0.06 ^c^	1.67 ± 0.05 ^d^
	SREBP-1c	1.08 ± 0.06 ^a^	1.23 ± 0.05 ^b^	1.58 ± 0.09 ^c^	1.78 ± 0.10 ^d^
	FAS	0.97 ± 0.05 ^a^	0.75 ± 0.06 ^b^	1.04 ± 0.09 ^a^	1.28 ± 0.07 ^c^
	ACC-1	0.96 ± 0.04 ^a^	0.73 ± 0.03 ^b^	1.28 ± 0.04 ^c^	1.26 ± 0.06 ^c^
	CPT-1	0.98 ± 0.04 ^a^	0.98 ± 0.04 ^a^	1.12 ± 0.07 ^a^	1.28 ± 0.08 ^b^
	GPR41	1.02 ± 0.06 ^a^	1.05 ± 0.06 ^a^	1.13 ± 0.12 ^a^	1.16 ± 0.13 ^a^
	GPR43	1.13 ± 0.07 ^a^	1.66 ± 0.15 ^b^	1.87 ± 0.11 ^c^	1.97 ± 0.16 ^c^
	AMPK-α	1.00 ± 0.06 ^a^	0.92 ± 0.07 ^a^	1.11 ± 0.07 ^a^	1.35 ± 0.04 ^b^
24 h	PPAR-γ	1.12 ± 0.05 ^a^	1.26 ± 0.04 ^b^	1.35 ± 0.07 ^b^	1.78 ± 0.10 ^c^
	C/EBP-α	1.08 ± 0.07 ^a^	1.18 ± 0.07 ^b^	2.63 ± 0.08 ^c^	3.69 ± 0.15 ^d^
	SREBP-1c	1.10 ± 0.09 ^a^	1.23 ± 0.08 ^b^	4.48 ± 0.10 ^c^	3.52 ± 0.12 ^d^
	FAS	0.94 ± 0.05 ^a^	0.78 ± 0.05 ^b^	0.67 ± 0.03 ^b^	0.55 ± 0.09 ^b^
	ACC-1	0.91 ± 0.05 ^a^	0.73 ± 0.03 ^b^	1.06 ± 0.08 ^a^	1.09 ± 0.06 ^a^
	CPT-1	0.93 ± 0.04 ^a^	0.76 ± 0.05 ^b^	1.26 ± 0.07 ^c^	1.37 ± 0.11 ^c^
	GPR41	1.04 ± 0.06 ^a^	1.09 ± 0.09 ^a^	1.13 ± 0.12 ^a^	1.15 ± 0.13 ^a^
	GPR43	1.18 ± 0.04 ^a^	1.68 ± 0.14 ^b^	1.96 ± 0.13 ^c^	2.07 ± 0.18 ^c^
	AMPK-α	0.92 ± 0.06 ^a^	0.77 ± 0.03 ^b^	0.73 ± 0.04 ^b^	0.53 ± 0.06 ^c^

Data are mean ± SD, and statistical significance was determined using a one-way analysis of variance (ANOVA) with Tukey’s multiple comparison test; *n* = 15/group. Different superscript lowercase letters in the same row indicate significant differences in relative mRNA expression levels between acetate concentrations (*p* < 0.05).

## Data Availability

All data has been presented in the text. Data sharing is not applicable to this article.
